# Why Are Clinicians Not Embracing the Results from Pivotal Clinical Trials in Severe Sepsis? A Bayesian Analysis

**DOI:** 10.1371/journal.pone.0002291

**Published:** 2008-05-28

**Authors:** Andre C. Kalil, Junfeng Sun

**Affiliations:** 1 Department of Internal Medicine, University of Nebraska Medical Center, Omaha, Nebraska, United States of America; 2 Assistant Professor, Department of Biostatistics, College of Public Health, University of Nebraska, Omaha, Nebraska, United States of America; Columbia University, United States of America

## Abstract

**Background:**

Five pivotal clinical trials (Intensive Insulin Therapy; Recombinant Human Activated Protein C [rhAPC]; Low-Tidal Volume; Low-Dose Steroid; Early Goal-Directed Therapy [EGDT]) demonstrated mortality reduction in patients with severe sepsis and expert guidelines have recommended them to clinical practice. Yet, the adoption of these therapies remains low among clinicians.

**Objectives:**

We selected these five trials and asked: Question 1-What is the current probability that the new therapy is not better than the standard of care in my patient with severe sepsis? Question 2-What is the current probability of reducing the relative risk of death (RRR) of my patient with severe sepsis by meaningful clinical thresholds (RRR >15%; >20%; >25%)?

**Methods:**

Bayesian methodologies were applied to this study. Odds ratio (OR) was considered for Question 1, and RRR was used for Question 2. We constructed prior distributions (enthusiastic; mild, moderate, and severe skeptic) based on various effective sample sizes of other relevant clinical trials (unfavorable evidence). Posterior distributions were calculated by combining the prior distributions and the data from pivotal trials (favorable evidence).

**Main Findings:**

Answer 1-The analysis based on mild skeptic prior shows beneficial results with the Intensive Insulin, rhAPC, and Low-Tidal Volume trials, but not with the Low-Dose Steroid and EGDT trials. All trials' results become unacceptable by the analyses using moderate or severe skeptic priors. Answer 2-If we aim for a RRR>15%, the mild skeptic analysis shows that the current probability of reducing death by this clinical threshold is 88% for the Intensive Insulin, 62–65% for the Low-Tidal Volume, rhAPC, EGDT trials, and 17% for the Low-Dose Steroid trial. The moderate and severe skeptic analyses show no clinically meaningful reduction in the risk of death for all trials. If we aim for a RRR >20% or >25%, all probabilities of benefits become lower independent of the degree of skepticism.

**Conclusions:**

Our clinical threshold analysis offers a new bedside tool to be directly applied to the care of patients with severe sepsis. Our results demonstrate that the strength of evidence (statistical and clinical) is weak for all trials, particularly for the Low-Dose Steroid and EGDT trials. It is essential to replicate the results of each of these five clinical trials in confirmatory studies if we want to provide patient care based on scientifically sound evidence.

## Introduction

*“If we begin with certainties, we shall end in doubts; but if we begin with doubts, and are patient with them, we shall end with certainties.”*

*Sir Francis Bacon (1605)*

More than 20 clinical trials involving over 10,000 patients have been performed in patients with sepsis and severe sepsis in the last 15 years with little success in reducing mortality [1]. More recently, five published clinical trials: Early Goal-Directed Therapy [2], Recombinant Human Activated Protein C [3], Low-Dose Steroid [4], Low-Tidal Volume-ARDS Network [5], and Intensive Insulin Therapy [6] demonstrated positive outcome results and brought the prospect of improving the survival of patients with severe sepsis.

Ten multinational medical societies sponsored a joint statement, ‘Surviving Sepsis Campaign’, in which recommendations are made to include the results of these trials in the standard of care for patients with severe sepsis [7]. These recommendations have also been evaluated by the Joint Commission on Accreditation of Healthcare Organizations [8]. Despite these positive outcomes and recommendations, scientists and clinicians have been either slow or resistant to adopt the results of these trials at face value in order to apply them to patient care [9]–[25]. Still, strong endorsement by the medical societies is not coming without criticisms and opposition by the medical community [8], [26]. Why is this resistance to accept statistically significant results from large clinical trials so accentuated in the sepsis field?

We propose that the genesis for most of these issues lies in the confounding interpretation and poor translation of these results to the bedside, and the lack of formal analysis combining previous evidence and the current positive clinical trials. While controversy is necessary for the progression of science [27], when it comes to treating a patient with severe sepsis, a clinical decision is also necessary for the betterment of this patient's outcome.

In the following paragraphs, we argue that the best solution for the understanding of pivotal clinical trials in severe sepsis can only come from a friendly reunion of classic (frequentist) and Bayesian statistical methodologies [28]–[31] **.** The application of this more inclusive and robust interpretation of trial results will facilitate their application directly to the bedside, and will hopefully further improve the care of our patients with severe sepsis. Moreover, this “dualistic” approach will also empower us to better define the need for confirmatory trials in order to optimize the current standard of care.

## Methods

### A. Methods Background

The “early goal-directed therapy” (EGDT) trial [2] will be used as a practical example to describe the rationale for our methodology. This trial aimed to compare the use of early volume replacement/vasopressor use in the treatment arm against standard of care in the control arm for patients with severe sepsis. The final results showed a 42% relative reduction in the risk of death (16% absolute risk reduction) of in-hospital mortality with a 95% confidence interval (CI) (0.13–0.62), p = 0.009. Common clinical interpretations are: (a) there is only a 0.9% probability of a false positive result (rejecting the null hypothesis when there is no treatment difference); (b) there is a 95% probability of the true risk ratio being somewhere between 0.13 and 0.62 (a reduction of 13–62% in the relative risk of death). These interpretations are incorrect due to the misunderstanding of the classic or frequentist (frequency based view of probability) statistical reporting used in this and most trials [32]–[35]. The correct interpretation of the classic method for this trial is the following: There is a 0.9% probability that results as good as or better than the ones found in this trial (42% relative reduction in the risk of death), will be observed among a large number of hypothetical repetitions of this trial under the null hypothesis of no treatment effect. In addition, the CIs generated from 95% of these hypothetical trials will cover the true mortality reduction. This rather convoluted language is the only possible interpretation which explicitly states that the classic method cannot provide the probabilities that clinicians are seeking for. In other words, clinicians are interpreting the conventional p values and CIs as ‘current probabilities’, although the classic method alone only provides us with probabilities over a large number of hypothetical repetitions in the long term.

Here is where the Bayesian methodology comes in to complement the classic interpretation of clinical trials. It allows us to think the way most clinicians are already thinking [36]! That is, what is the posterior or ‘current probability’ (not the probability in the long term of hypothetical trial repetitions) of observing this outcome in a given trial or population.

In order to use the Bayesian methodology, a prior probability is required. The prior probability can be based on all available evidence (i.e. biological rationale and its pre-clinical evaluation; observational and experimental clinical data) gathered by studies other than the new trial being currently analyzed. An analogy to the diagnostic setting states that “it is not possible to find a probability of having a disease based on tests results without specifying the disease's prevalence” [37]. The basic idea described by Bayes demonstrates that the product between the prior probability and the evidence provided by the new trial (also called Bayes factor) will give us the posterior probability, which we call the “current probability” in our paper. This simple and clever algebraic calculation allows us to overcome the unsolvable ‘long term hypothetical repetitions’ inherent issue of the classic method. At the same time, it gives us what we are mostly looking for in our daily medical practice, i.e. what is the current probability to achieve the results of this pivotal trial in my patients? The potential “subjectivity” of these priors have made some statisticians and clinicians concerned about the use of this method. However, the complete exclusion of prior knowledge and evidence from the design and interpretation of recently completed clinical trials has been compared to a sentencing judge who overlooks the prior convictions of a habitual criminal [38]. Needless to say, the commonly used classic methodology in current clinical trials is far from objective. For example, the models assumed, the parameters and hypothesis chosen, and the experimental designs employed [37] are typical features that incorporate much subjectivity into the classic analysis. We agree with Berry [37] that “…silent subjectivities such as these (seen with classic methods) are dangerous in that they are difficult or impossible to make explicit. By contrast, subjectivity in prior distributions (as seen with Bayesian methods) is explicit and open to examination-and critique-by all”. Thus, how should we best determine the priors for this study? This so-called subjectivity is easily resolved in the severe sepsis world because we have many negative clinical trials done before the current positive trials, which set the stage for the perfect use of Bayesian methodology. We will provide the clinician with a realistic spectrum of prior distributions, so he/she can find the current probability of the new treatment being no better than the control (standard of care), and the current probability of reducing mortality by a clinically meaningful threshold. These two probabilities will allow the clinician to make the best clinical decision for the patient with severe sepsis without being entirely dependent on sponsors, regulators, editors, and experts in the field.

### B. Methods Description

#### 1. What is the current probability that the new therapy is not better than the standard of care in my patient with severe sepsis?

The goal of the first part of our study is to assess the current probability that the new therapy is no better than the control, i.e. standard of care. We used the log-odds ratio (ln(OR)) of death for the new treatment group compared to the control group. We considered the new therapy to be no better than the control if the ln(OR) was found to be greater than −0.05 [38], [39]. We then constructed various prior distributions of ln(OR) assuming previous trials of different effective sample sizes (see Appendix S1). The prior distributions may be based on the following information: Early Goal-Directed Therapy [40]–[47], rhAPC [48]–[53], Low-Dose Steroids [54]–[67], Low-Tidal Volume [68]–[74], Intensive Insulin Therapy [75]–[85]. Of note, some trials were performed before and others after the pivotal positive trial of a given therapy. This inclusive approach of all evidence available is an important strength of the Bayesian technique, which does not require that the priors have a temporal order [32], [37], [86], [87]. This list of studies for each therapy allows the reader to get his/her own effective sample size by summing up the prior unfavorable evidence (total sample size of relevant negative clinical trials). If believing there is no relevant previous negative data, one can assume the effective sample size to be 1 [88]. This gives us the non-informative prior, which is our ‘enthusiastic’ prior, since it is ignoring all negative sepsis trials already published. If one is skeptical about the effects of the new therapy based on previous phase 2 and 3 trials, observational studies, or clinical experience, the effective sample size of negative evidence for the (skeptical) prior will be larger. Thus, the more skeptical one is based on the prior information, the harder it is to conclude efficacy using the same pivotal trial data. Based on the mortality rate and the required sample size to evaluate new therapies for severe sepsis in current times, the following effective sample sizes of negative evidence were analyzed for each new therapy: Enthusiastic (n = 1); Mildly skeptic (n = 200); Mild-moderately skeptic (n = 500); Moderately skeptic (n = 1,000); Severely skeptic (n = 2,000). We consider the current probability of ln(OR) >−0.05 being less than 0.05 as sufficient evidence to conclude that the new treatment is better than the control. For example, if the clinician sums up approximately 500 subjects from 3 previous negative trials on low-tidal volume therapy [69]–[71] (i.e. mild to moderate skeptic about this therapy), our analysis (table 1) will provide a 0.05 current probability of low-tidal volume therapy being no better than control. As the reader can appreciate in table 1, because of the negative prior information that needs to be overcome, this current probability (0.05) is different from the classic p value reported in the original trial publication (p = 0.007).

**Table 1:** Current probability of new treatment being no better than control based on mortality outcomes.

CLINICAL TRIALS (Favorable Evidence)	Early Goal- Directed Therapy	Activated Protein C	Activated Protein C (APACHE II >25)	Low-Dose Steroids (Non-Responders)	Low-Tidal Volume	Intensive Insulin Therapy (>5 days in ICU)
**Published P values** (Frequentist)	**0.009**	**0.005**	**0.0002**	**0.02**	**0.007**	**0.005**
**Sample Size of Unfavorable Evidence** (Bayesian Priors)	**CURRENT PROBABILITY OF NEW TREATMENT BEING NO BETTER THAN CONTROL**					
**ENTHUSIASTIC** (Sample Size = 1)	**0.01**	**0.009**	**0.0003**	**0.03**	**0.009**	**0.003**
**MILD SKEPTIC** (Sample Size = 200)	**0.05**	**0.02**	**0.002**	**0.10**	**0.02**	**0.02**
**MILD-MODERATE SKEPTIC** (Sample Size = 500)	**0.14**	**0.03**	**0.007**	**0.21**	**0.05**	**0.05**
**MODERATE SKEPTIC** (Sample Size = 1000)	**0.25**	**0.05**	**0.03**	**0.33**	**0.10**	**0.12**
**SEVERE SKEPTIC** (Sample Size = 2000)	**0.41**	**0.12**	**0.09**	**0.47**	**0.21**	**0.23**

#### 2. What is the current probability of decreasing the relative risk of death of my patient with severe sepsis by a meaningful clinical threshold?

Based on the multitude of clinical trials already published [1], the recent FDA approval of a new therapy on sepsis [3], and a 28-day mortality ranging from 30–40%, no new therapy for severe sepsis will likely be accepted by clinicians or regulators if the relative risk reduction (RRR) for mortality is not greater than 15–25% (absolute risk reduction (ARR) ≥5–10%). For example, a trial with a control arm mortality of 30% and a experimental arm mortality of 25% would result in an ARR = 5% and a RRR = 16%. Thus, we will present the current probabilities for greater than 15%, 20%, and 25% RRR for mortality in each trial (see Appendix S1). This second analysis can be thought of as analogous to ‘clinical significance’. For example, if the clinician sums up approximately 1000 subjects from 2 previous negative trials on volume replacement/vasopressor use [45], [46] (i.e. moderate skeptic about this therapy) and believes that a minimum of 15% RRR needs to be demonstrated to add the EGDT to standard of care of patients with severe sepsis, our analysis (table 2) will provide a 6% current probability of reaching at least this RRR in mortality. This current probability (6%) of reducing mortality by at least 15% is different from the classic overall RRR reported in the original trial publication (42%). This level of prediction can not be achieved with classic methods alone.

**Table 2:** Probability of Relative Risk Reduction (RRR) of Mortality by EGDT.

EARLY GOAL-DIRECTED THERAPY			
SAMPLE SIZE OF UNFAVORABLE EVIDENCE	CURRENT PROBABILITY OF RELATIVE RISK REDUCTION		
	RRR>15%	RRR>20%	RRR>25%
**ENTHUSIASTIC**	94%	87%	78%
**MILD SKEPTIC**	62%	41%	22%
**MILD-MODERATE SKEPTIC**	27%	9%	2%
**MODERATE SKEPTIC**	6%	1%	0
**SEVERE SKEPTIC**	0.4%	0	0

The results of the five positive clinical trials [2]–[6] will be discussed in the context of the two probability questions described above. Because of the non-significant overall results of the low-steroid trial for mortality [4], we will evaluate the prospectively defined “non-responders” sub-population. Due to the low overall control mortality (8%), and the inclusion of all comers (with and without severe sepsis) to a surgical ICU in the intensive insulin trial [6], we will evaluate the prospectively defined “>5 days in ICU” sub-population. This subgroup control had mortality rates (20%) closer to those of the other trials. The rhAPC trial [3] will have two analyses, since the drug was FDA approved based on the sicker (APACHE II >25) sub-population. Even though the ARDSNet Low-Tidal Volume trial [5] was not specifically designed for patients with severe sepsis, we included it because approximately 60% of the trial population had sepsis or pneumonia, and the trial results have been recommended for patients with severe sepsis by practice guidelines [7].

## Results

### 1. What is the current probability that the new therapy is not better than the standard of care in my patient with severe sepsis?

Table 1 shows the current probability of the new treatment being no better than the control. If we are enthusiastic about the five trials, all probabilities are small. If we are just mildly skeptic, the probabilities from the EGDT [2] and Low-Dose Steroid [4] trials become 0.05 or larger, which may not provide strong enough evidence to change the standard of care. If we take into account the preceding multitude of unfavorable trials and analyze these results in the light of a mild-moderate skeptical view, the current probabilities for these two trials rise to 0.14 and 0.21, respectively. The results of rhAPC, Low-Tidal Volume, and Intensive Insulin trials remain acceptable if we assume a mild skepticism-all with current probabilities of less than 0.05. The mild-moderate skeptic analysis makes both the Low-Tidal Volume and Intensive Insulin results reach the 0.05 probability of the new treatment being no better than the control, and the moderate skeptic analysis shows the rhAPC trial with the same 0.05 probability as well. The rhAPC APACHE II sub-analysis shows probabilities below 0.05 in all prior levels except in the severe skeptic analysis. If the clinician is severely skeptical, no trial results will lead to changes in the standard of care.

### 2. What is the current probability of decreasing the relative risk of death of my patient with severe sepsis by a meaningful clinical threshold?

Table 2 illustrates the current probabilities of achieving at least a specific clinically meaningful RRR based on the chosen cut-off and prior distribution. For a clinician who is enthusiastic (i.e. choosing the enthusiastic prior) about EGDT and believes that an RRR should not be less than 20% to use this new therapy in patient care, the chance of EGDT to decrease the relative risk of death more than 20% is 87%. If RRR>15% is the goal, this therapy will have a 94% (enthusiastic) and 62% (mildly skeptic) current probabilities. This is a tangible and easy result to translate and apply to bedside. For the clinician who is mildly skeptical about this therapy, the chance of EGDT to decrease the relative risk of death more than 20% in a given patient will decrease to 41%, which may be too low for general clinical application. If a moderately skeptical prior is applied to this trial, the probability of having a RRR greater than 15% and 20% drops substantially to 6% and 1%, respectively.

For the rhAPC overall results (table 3), in the best case scenario (enthusiastic), the current probability of reaching a 20% RRR in mortality is 43%. If the clinician is comfortable with a 15% RRR, then the probability of reaching this cut-off with rhAPC goes up to 75% for the enthusiastic, and to 65% for the mildly skeptical, but remains low at 30% for the moderately skeptical. On the other side, if we analyze this trial based on APACHE II >25 subgroup and enthusiastic prior, then there is 97%, 90%, and 72% probability of respectively reaching RRR of 15%, 20%, and 25% with rhAPC. These are more optimistic results than the overall trial analysis, but if the clinician remains a mild-moderate skeptic, the probability of achieving any of those same RRR thresholds becomes smaller; 63%, 30%, and 7%, respectively. The moderate and severe skeptic analyses show most probabilities of reaching any meaningful RRR in the single digits.

**Table 3 pone-0002291-t003:** Probability of Relative Risk Reduction (RRR) of Mortality by recombinant human APC.

ACTIVATED PROTEIN C			
SAMPLE SIZE OF UNFAVORABLE EVIDENCE	CURRENT PROBABILITY OF RELATIVE RISK REDUCTION		
	RRR >15%	RRR >20%	RRR >25%
**ENTHUSIASTIC**	75%	43%	14%
**MILD SKEPTIC**	65%	31%	7%
**MILD-MODERATE SKEPTIC**	50%	17%	3%
**MODERATE SKEPTIC**	30%	6%	0.4%
**SEVERE SKEPTIC**	9%	0.5%	0

ACTIVATED PROTEIN C (APACHE II >25)			
SAMPLE SIZE OF UNFAVORABLE EVIDENCE	CURRENT PROBABILITY OF RELATIVE RISK REDUCTION		
	RRR >15%	RRR >20%	RRR >25%
**ENTHUSIASTIC**	97%	90%	72%
**MILD SKEPTIC**	88%	66%	35%
**MILD-MODERATE SKEPTIC**	63%	30%	7%
**MODERATE SKEPTIC**	27%	5%	0.2%
**SEVERE SKEPTIC**	3%	0.1%	0

The Low-Dose Steroid trial analysis demonstrates well the importance of this type of clinical threshold analysis. In the best case-scenario for the least meaningful RRR (15%), the enthusiastic approach shows a current probability of 58% (table 4). Even for the mildly skeptic, steroids have an unacceptably low current probability (20% or less) of reaching any meaningful RRR in mortality. We also performed an additional analysis about the probability of steroids reaching a RRR>10%, but except for the enthusiastic prior (76%), all other probabilities remain similarly poor (41% or less).

**Table 4 pone-0002291-t004:** Probability of Relative Risk Reduction (RRR) of Mortality by Low-Dose Steroid.

LOW-DOSE STEROID			
SAMPLE SIZE OF UNFAVORABLE EVIDENCE	CURRENT PROBABILITY OF RELATIVE RISK REDUCTION		
	RRR >15%	RRR >20%	RRR >25%
**ENTHUSIASTIC**	58%	37%	19%
**MILD SKEPTIC**	17%	4%	0.5%
**MILD-MODERATE SKEPTIC**	3%	0.1%	0
**MODERATE SKEPTIC**	0.1%	0	0
**SEVERE SKEPTIC**	0	0	0

The Low-Tidal Volume trial (table 5) shows a current probability of 83% (enthusiastic) or 65% (mild skeptic) for the physician who is looking for a RRR>15%. However, for the mildly skeptic requiring a RRR>20% the probability drops to 37%, and for the mild-moderate skeptic requiring any RRR, the probabilities of benefit from this therapy remain all below 40%.

**Table 5 pone-0002291-t005:** Probability of Relative Risk Reduction (RRR) of Mortality by Low-Tidal Volume.

LOW-TIDAL VOLUME			
SAMPLE SIZE OF UNFAVORABLE EVIDENCE	CURRENT PROBABILITY OF RELATIVE RISK REDUCTION		
	RRR >15%	RRR >20%	RRR >25%
**ENTHUSIASTIC**	83%	62%	34%
**MILD SKEPTIC**	65%	37%	13%
**MILD-MODERATE SKEPTIC**	41%	14%	2%
**MODERATE SKEPTIC**	16%	2%	0.1%
**SEVERE SKEPTIC**	2%	0	0

The Intensive Insulin trial (table 6) shows consistent probabilities of reducing the risk of death (69–98%) for all RRR levels in both enthusiastic and mild skeptic analyses. The probability remains above 60% even in the mild-moderate skeptic analysis if the aim is a RRR>15%. However, for the moderate or severe skeptic approach, all results are below 42%.

**Table 6:** Probability of Relative Risk Reduction (RRR) of Mortality by Intensive Insulin Therapy.

INTENSIVE INSULIN THERAPY			
SAMPLE SIZE OF UNFAVORABLE EVIDENCE	CURRENT PROBABILITY OF RELATIVE RISK REDUCTION		
	RRR >15%	RRR >20%	RRR >25%
**ENTHUSIASTIC**	98%	96%	93%
**MILD SKEPTIC**	88%	80%	69%
**MILD-MODERATE SKEPTIC**	68%	52%	35%
**MODERATE SKEPTIC**	42%	23%	9%
**SEVERE SKEPTIC**	16%	4%	0.6%

## Discussion

The first analysis indicates that there is sufficient evidence to support the efficacy in all five trials only if we are enthusiastic with respect to the prior distribution of each of these therapies. If we analyze them with the mild skepticism, only the Intensive Insulin, rhAPC, and Low-Tidal Volume trials show beneficial results. Although the rhAPC APACHE II >25 sub-analysis remains beneficial in the moderate skeptic analysis, the original results have not been validated in a prospective phase III trial yet. Based on our current results indicating high probability of new treatment being no better than controls in all skeptic analyses for the EGDP and Low-Dose Steroid trials, as well as on the numerous unfavorable trials evaluating different regimens of volume replacement/vasopressors or steroids in severe sepsis, we demonstrate that the beneficial results from these two trials are the ones with the weakest strength of evidence. All five trials become unacceptable if we are moderately or severely skeptical, because the current probability of the new therapy being no better than the control is too high for general clinical application. The fact that most overall beneficial results are not even that impressive with the mild-moderately skeptic analysis is concerning.

The second analysis based on a specific clinical cut-off of the RRR brings important light to the interpretation of these trials. Assuming an enthusiastic prior, the Intensive Insulin, the rhAPC APACHE II >25, and the EGDP trials demonstrate the highest probability (87–96%) of reaching an RRR of at least 20% for mortality. However, the absence of a majority of patients with severe sepsis in the Intensive Insulin trial, the absence of prospective validation of the rhAPC APACHE II >25 subgroup population, and the weak strength of evidence from our first analysis for the EGDP trial, all suggest that more skepticism should be taken before assuming these probabilities of RRR>20%. In this case, the moderate skeptic analysis for these 3 trials shows all current probabilities of 23% or less of reducing death risk by more than 20%. On the other hand, if we are mildly skeptical and aim a RRR>15%, the probability of reducing the risk of death is 88% for the Intensive Insulin trial, 62–65% for the rhAPC, Low-Tidal Volume, and EGDT trials, and just 17% for the Low-Dose Steroid trial. Unless we are less ambitious with respect to the RRR, i.e. 15%, and accept an enthusiastic to mildly skeptic prior, none of these trials have shown strong enough evidence for diminishing the risk of death in patients with severe sepsis. Of note, the consistently very low current probabilities (poor strength of evidence) of observing mortality reduction with Low-Dose Steroids in both of our analyses (questions 1 and 2) was just confirmed by a recently published phase III trial [66]. Interestingly, our statistical analysis predicting the poor results of Low-Dose Steroids was completed and submitted long before the report of this new phase III trial.

We would like to recognize some limitations of our study. The statistical approach we used for this study may appear to produce more conservative results than conventional methods, but this is, in fact, the main strength of our data. The Bayes' theorem is uncontroversial if derived from known data [88]. We strongly believe that the prior negative evidence is so abundant that we have the ethical obligation to consider and use this methodology in the analysis of any new therapy for severe sepsis. The different sample sizes of each trial may have influenced the current probabilities of our first analysis, but the consistent results found in both first and second analyses make the sample size influence less likely. Also, because some of the therapeutic interventions were not identical within a specific class, and other studies were different with respect to their trial design, we advise the reader to carefully evaluate the most appropriate priors to avoid overt pessimistic or optimistic current probabilities.

In conclusion, our study provides four clinical and research lessons with profound implications to the care of our patients with severe sepsis:

### Lesson 1–Need for confirmatory trials

Our results unambiguously demonstrate that it is important to replicate the results of each of these trials in well designed confirmatory studies if we want to provide patient care based on scientifically sound evidence. Further, the many study design issues raised, e.g. standard of care of control groups (EGDT and Low-Tidal Volume trials [10]–[12], [17], [21]–[24]; the rhAPC APACHE II >25 subgroup analysis without prospective validation [3] and the poor results of this subgroup in the ADDRESS trial [49], [89]; the controversial definitions of adrenal insufficiency [20], [90], [91], and the just reported Low-Dose Steroid phase III trial with negative results [66]; and the failure of the Intensive Insulin Therapy to improve survival in the medical ICU population [75] all corroborate our conclusion for lesson 1.

### Lesson 2–Standard of care for patients with severe sepsis

The strength of evidence (statistical and clinical) is overall weak for the five trials. These results make any legitimate changes in the standard of care a very difficult task to accomplish. While we endorse the genuine need for more evidence, we are aware of the urgent need to improve the survival outcome of our patients with severe sepsis. How to reconcile this apparent conundrum? Sir Austin Hill already had the answer in his seminal paper from 1965: “All scientific work is incomplete–whether it be observational or experimental. All scientific work is liable to be upset or modified by advancing knowledge. That does not confer on us a freedom to ignore the knowledge that we already have, or to postpone the action that it appears to demand at a given time” [92]. Before we have the results of confirmatory trials, we urge clinicians to use our clinical threshold analysis of RRR for mortality from each trial (tables 2–6) to guide the best course of action to take for their patients with severe sepsis.

### Lesson 3–No need for changes in the measurement of ICU quality of care

The most important premise to change the measurement of quality of care must be based on strong and established scientific evidence, but this is lacking at this time. When we completed our study it became obvious that, with such low strength of evidence and critical need for confirmatory trials, none of these studies' results should be applied as rigid tools to measure quality of care in patients with severe sepsis in the ICU.

### Lesson 4–Need for both classic and Bayesian interpretation of clinical trials

As we demonstrate in this paper, the dual use of these methods is powerful and synergistic to accomplish an ample interpretation of these pivotal trials. We strongly suggest that trialists, sponsors, regulators, and journal editors become more proactive with respect to the use of this dual statistical approach. Patients will be the ultimate beneficiaries from this more encompassing strategy.

We anticipate that our comprehensive analysis and interpretation of these trials will bestow realistic and practical tools to clinicians to decide on their own and without undue influence how to best apply the results of these trials to their patients with severe sepsis.

## Supporting Information

Appendix S1 (0.05 MB DOC)
