# Supplementary material for: Why Are Clinicians Not Embracing the Results from Pivotal Clinical Trials in Severe Sepsis? A Bayesian Analysis
Source: PLoS One. 2008 May 28;3(5):e2291. doi: 10.1371/journal.pone.0002291 (PMC2384005; doi:10.1371/journal.pone.0002291)
Supplement: Appendix S1 — (0.05 MB DOC) [file pone.0002291.s001.doc]

**APPENDIX S1**

**METHODS:**

**A. Methods Description:**

1. **What is the current probability that the new therapy is not better than the standard of care in my patient with severe sepsis?**

Let *θ*_1_ be the ln(OR) of death for the new treatment group compared to the control group. We used the estimate of *θ*_1_ and its variance ($\hat{\theta}_{1}$ and $\hat{\sigma}_{1}^{2}$) based on adjusted OR (provided by logistic regression) whenever possible. Otherwise, let *a* and *b* be the numbers of death, *c* and *d* be the numbers of patients who survived in the treatment group and the control group, respectively. Let $n_{T}=a+c,n_{C}=b+d.$ It is well-known that the estimate of *θ*_1_ and its variance are $\hat{\theta}_{1}=\ln\left(\frac{ad}{bc}\right)$ and $\hat{\sigma}_{1}^{2}=\frac{1}{a}+\frac{1}{b}+\frac{1}{c}+\frac{1}{d}$, and $\hat{\theta}_{1}$ has approximate normal distribution with mean *θ*_1_ and variance$\hat{\sigma}_{1}^{2}$ [93]. Assume the prior distribution of *θ*_1_ be normal with mean 0 and variance *σ_p_*_1_^2^. Then it is easy to show [94] that the posterior distribution of *θ*_1_ is

(1)

We can then calculate the posterior probability of the new treatment being no better than the control (ln(OR) > -0.05).

The prior distribution was chosen based on the effective sample size of prior clinical trials. Similar to Spiegelhalter, et al [94], we assume the hypothetical prior trial have equal sample size *n* and equal event rate *p* in both arms. Then it is easy to show that

$\sigma_{p1}^{2}\approx\frac{2}{np(1-p)}.$ (2)

Replacing *p* with the observed overall mortality rate for the control arm of the current trial, we can specify *σ_p_*_1_^2^ based on the effective sample size (2*n*) of prior trials.

1. **What is the current probability of decreasing the relative risk of death of my patient with severe sepsis by a meaningful clinical threshold?**

We consider the log-transformed relative risk (ln(RR)) of death for the treatment group compared to the control group, calculated from the observed numbers of deaths from each trial. The parameter of interest *θ*_2_ = ln(RR). The estimate of *θ*_2_ and its variance based on the data are $\hat{\theta}_{2}=\ln\left(\frac{an_{C}}{bn_{T}}\right)$ and $\hat{\sigma}_{2}^{2}=\frac{c}{an_{T}}+\frac{d}{bn_{C}}$, and$\hat{\theta}_{2}$ has approximate normal distribution with mean *θ*_2_ and variance$\hat{\sigma}_{2}^{2}$ for large trials [93]. Assume the prior distribution of *θ*_2_ be normal with mean 0 and variance *σ_p_*_2_^2^. Similar to the first analysis, we defined non-informative and skeptic prior distributions based on the effective sample sizes of prior trials. Again assuming the prior trial have equal sample size *n* and equal event rate *p* in both arms, it is easy to show that

$\sigma_{p2}^{2}\approx\frac{2(1-p)}{np}.$ (3)

Replacing *p* with the observed mortality rate for the control arm of the current trial, we can specify *σ_p_*_1_^2^ based on the effective sample size (2*n*) of prior trials. We then derive the posterior distribution of *θ*_2_ from Equation (1), using instead of . Then we can easily calculate the posterior probability of RRR being greater than a specific clinically meaningful cut-off.
